# Effect of Dl-3-n-Butylphthalide on olfaction in rotenone-induced Parkinson’s rats

**DOI:** 10.3389/fneur.2024.1367973

**Published:** 2024-04-08

**Authors:** Jiawei Wang, He Li, Canran Wang, Dayong Li, Yong Zhang, Meichan Shen, Xiangdong Xu, Tong Wu

**Affiliations:** ^1^The First Clinical Medical College, Shandong University of Traditional Chinese Medicine, Jinan, China; ^2^Third Department of Neurology, Affiliated Hospital of Shandong University of Traditional Chinese Medicine, Jinan, China; ^3^Geriatrics Department, Yuncheng County Traditional Chinese Medicine Hospital, Heze, China

**Keywords:** Parkinson’s disease, Butylphthalide, olfaction, rotenone, rats

## Abstract

**Background:**

Parkinson’s disease (PD) is the second most common neurodegenerative disease after Alzheimer’s disease. Olfactory dysfunction (OD) is an important nonmotor feature of PD. Dl-3-n-Butylphthalide (NBP) is a synthetic compound isolated from *Apium graveolens* seeds. The present study was conducted to investigate the effect of NBP on olfaction in rotenone-induced Parkinson’s rats to explore the mechanism and pathway of OD in PD.

**Methods:**

The PD model was established using rotenone-induced SD rats, divided into blank control, model, and treatment groups. A sham group was also established, with 10 rats in each group. The treatment group was given NBP (1 mg/kg, 10 mg/kg, and 100 mg/kg, dissolved in soybean oil) intragastrically for 28 days. Meanwhile, the control group rats were given intra-gastrically soybean oil. After behavioral testing, all rats were executed, and brain tissue was obtained. Proteomics and Proteomic quantification techniques (prm) quantification were used to detect proteomic changes in rat brain tissues.

**Results:**

Compared with the control group, the model group showed significant differences in behavioral tests, and this difference was reduced after treatment. Proteomics results showed that after treatment with high-dose NBP, there were 42 differentially expressed proteins compared with the model group. Additionally, the olfactory marker (P08523) showed a significant upregulation difference. We then selected 22 target proteins for PRM quantification and quantified 17 of them. Among them, the olfactory marker protein was at least twofold upregulated in the RTH group compared to the model group.

## 1 Introduction

Parkinson’s disease (PD) is the most common neurodegenerative disease, affecting about 1% of people over the age of 60 (1). Unfortunately, with the aging of the population, the prevalence of Parkinson’s disease is increasing (2). PD could cause movement disorders and olfactory dysfunction in patients, making patients lose the pleasure of eating and the ability to recognize dangerous environments (3). Olfactory dysfunction seriously affects the quality of life of patients (4). Our treatment options for non-movement disorders, such as olfactory, are still limited (5). Thus, unearthing and testing new treatments for olfactory disorders in PD still has a long way to go.

Dl-3-n-Butylphthalide (NBP) is a synthetic compound based on l-3-n-Butylphthalide isolated from *Apium graveolens* seeds (6). Pharmacological studies have demonstrated that NBP has a series of mechanisms of action, such as rebuilding microcirculation, protecting mitochondrial function, inhibiting oxidative stress, and inhibiting neuronal apoptosis (7). Previous studies have also confirmed that NBP can improve behavioral function without significant side effects (8, 9). Additionally, animal experiments have demonstrated that NBP stimulates the peripheral olfactory system of *Stegobium paniceum* and causes positive chemotaxis in adult drugstore beetles (10). Furthermore, NBP has therapeutic potential for improving PD behavior, olfactory function, and other functions (11). However, there are few studies on the improvement of PD olfactometry. Therefore, we designed animal and proteomics experiments to explore the effect of NBP on the olfactometry of rotenone-induced Parkinson’s rats.

## 2 Materials and methods

### 2.1 Materials

#### 2.1.1 Experimental drugs

NBP was provided free of charge by Shijiazhuang Pharmaceutical Group NBP Pharmaceutical Co., LTD. (Shijiazhuang, China).

#### 2.1.2 Experimental animals

Male SD rats (8 weeks of age, 250–300 g). All animal experiments were pre-approved by the Experimental Animal Ethics Committee of the Affiliated Hospital of Shandong University of Chinese Medicine (approval number: LAEC-2020-228).

#### 2.1.3 Main reagents: rotenone

Cell counting Kit 8 (CCK-8) and Reactive Oxygen Species (ROS) assay kit were obtained from Beyotime Biotechnology Co., Ltd. (Nantong, China). The Annexin V-FITC Apoptosis Detection Kit and Hoechst 33258 dye were purchased from Kagan Biotechnology Co., LTD. (Nanjing, China). The BCA protein assay reagent and RIPA lysis buffer were obtained from Thermo Fisher Technologies (MA, United States). Anti-tyrosine hydroxylase (TH, ab137869 and ab113), anti-alpha-synaptic nucleoprotein (a-Syn, ab138501), anti-alpha-synaptic nucleoprotein (phosphorylated S129) (p-a-Syn, ab51253), anti-polyadp ribose polymerase 1 (PARP1, ab191217), anti-allogenic inflammatory factor 1 (IBA1, ab178847) antibody, donkey anti-rabbit IgG (Alexa Fluor 488) (ab150073), donkey anti-rabbit IgG (Alexa Fluor 647) (ab150075), and donkey anti-sheep IgG (Alexa Fluor 647) (ab150179) were purchased from Abcam (Cambridge, United States). Antiapoptoses-associated speck-like proteins containing antibodies against CARD (ASC, 67824), poly/monadp ribose (PAR, 83732), and phosphorylated histone H2AX (Ser139) (pgH2AX, 9718) were purchased from Cell Signaling Technology, Inc. (Beverly, United States). The anti-Nacht, LRR, and PYD domains contained protein 3 (NLRP3, 19771-1-AP), anti-trypsin 1/p20/p10 (Caspase 1, 22915-1-AP), anti-interleukin-1β (IL-1b, 16806-1-AP), anti-glial fibrillary acid protein (GFAP, 16825-1-AP), anti-β-actin (β-actin, 60008-1-Ig), goat anti-rabbit IgG (SA00001-2), and goat anti-mouse IgG (SA00001-1) were purchased from Proteintech Group (Chicago, Illinois, USA). Urea, iodoacetamide, and DL-Dithiothreitol were obtained from Sigma-Aldrich. Trypsin was from Promega, acetonitrile was from ThermoFisher Scientific, and the protease inhibitor cocktail was from Merck Millipore.

### 2.2 Methods

#### 2.2.1 Grouping and intervention methods

The animals were domesticated in standard facilities for 7 days in the experiment. Drug concentrations were selected based on previous reports (11). Some previous studies have provided clues to using experimental concentrations of NBP (9, 10). Combined with our pilot study, the three comprehensive considerations of 1 mg/kg (low), 10 mg/kg (low), and 100 mg/kg (high) were applied in this study. Sixty male rats were randomly divided into six groups. They were: (1) MA group (without any treatment), (2) sham group (intraperitoneal injection of 98% sunflower oil 1 mL/kg and 2%DMSO), (3) RT + SALINE group (4) RT + NBP low dose group (1 mg/kg), (5) RT + NBP medium dose group (10 mg/kg) and (6) RT + NBP high dose group (100 mg/kg). RT group rats were intraperitoneally injected with rotenone (2.75 mg/kg) for 14 days. The treatment group was given intragastric administration of NBP (1 mg/kg, 10 mg/kg, and 100 mg/kg, dissolved in soybean oil) for 28 days. Simultaneously, control rats were intraperitoneally given soybean oil. All rats were executed after behavioral testing, and brain tissue was obtained.

#### 2.2.2 Behavioral assessments

##### 2.2.2.1 Open field test

The subject rats were placed in a white square container (40 × 40 cm^2^), and a video camera was mounted in the top center of the box to record the behavior of the rats for 5 min. The center and peripheral areas were mapped using an automatic recording tracking system, and the total distance traveled by the subject mice was calculated.

##### 2.2.2.2 Rotarod test

The rotarod test was performed using a rotarod treadmill. The rats were placed on the rotarod and tested in a constant acceleration mode from an initial 5 rpm/min to 40 rpm/min over 5 min. Then, we recorded the maximum time each rat stayed on the rod. The test was repeated thrice with an interval of 20 min and the average dwell time of the three tests was recorded.

##### 2.2.2.3 Suspension test

The rats were suspended from a horizontal nylon line 20 cm off the ground. The time that the rats could grasp the nylon rope with one paw was recorded.

##### 2.2.2.4 Buried pellet test

Before testing, we recorded the weight of each rat and then restricted food intake to 90% of body weight. Prior to testing and during food restriction, we gave each rat 1–2 pieces of the pellets to be used during the test (a piece of sweetened cereal). We filled a clean rat cage ~3 cm high with clean bedding, ensuring the bedding is evenly distributed throughout the cage. We then set a timer for 5 min. We buried 1 sweetened cereal pellet 0.5 cm below the bedding so it is not visible. Then, we removed the test rat from its home cage, placed it in the test cage’s center, and started the timer. We stopped the timer when the rat uncovered the pellet and began eating it. Furthermore, we recorded the time it took for the rats to find and start eating the pellets.

#### 2.2.3 Proteomics

##### 2.2.3.1 Protein extraction

The samples were removed from −80°C, weighed into a pre-cooled mortar with liquid nitrogen, and ground to powder with liquid nitrogen. Each group of samples received 4 times the volume of powder lysis buffer (8 M urea, 1% protease inhibitor), in which the samples were then sonicated and lysed. At 4°C, the samples were centrifuged at 12,000 g for 10 min to remove cell debris. Moreover, the supernatant was transferred to a new centrifuge tube for protein concentration determination using the BCA kit.

##### 2.2.3.2 Trypsin digestion

We took equal amounts of each sample protein for enzymatic digestion and adjusted the volume with lysis solution. Dithiothreitol (DTT) was added to a final concentration of 5 mM and reduced for 30 min at 56°C. Iodoacetamide (IAA) was added to a final concentration of 11 mM, incubated for 15 min at room temperature, and protected from light.

##### 2.2.3.3 Liquid chromatography-mass spectrometry analysis

Peptides were dissolved with liquid chromatography mobile phase A and separated using a Nano Elute ultra-performance liquid chromatography system. The ion source voltage was 2.3 kV, and the FAIMS compensation voltage (CV) was set to −45 V and −65 V. The peptide parent ions and their secondary fragments were detected and analyzed using a high-resolution Orbitrap.

##### 2.2.3.4 Independent validation of top-up-expressed proteins by PRM method

LC–MS analysis was performed following protein extraction and enzymatic digestion using the above method. The mobile phase A was an aqueous solution containing 0.1% formic acid and 2% acetonitrile; the mobile phase B was an acetonitrile-water solution containing 0.1% formic acid. The ion source voltage was set at 1.80 kV, and the peptide parent ions and their secondary fragments were detected and analyzed using time of flight. The secondary mass spectrometry scan range was set to 100–1,700. The mass error tolerance of the primary parent ion was set to 20 ppm for the first search and 20 ppm for the main search, respectively. The mass error tolerance of the secondary fragment ion was set to 20 ppm.

#### 2.2.4 Statistical analysis

All results were analyzed using GraphPad Prism 9.0.2 (GraphPad Software), and data were shown as mean ± standard error of the mean (SEM). One-way analysis of variance (ANOVA) with Tukey’s test for *post hoc* comparisons was used to define the differences between groups. Data are representative of at least three independent experiments. A *p*-value < 0.05 was considered statistically significant.

## 3 Results

### 3.1 NBP improved rotenone-induced behavioral impairment in rats

**Figure 1:**
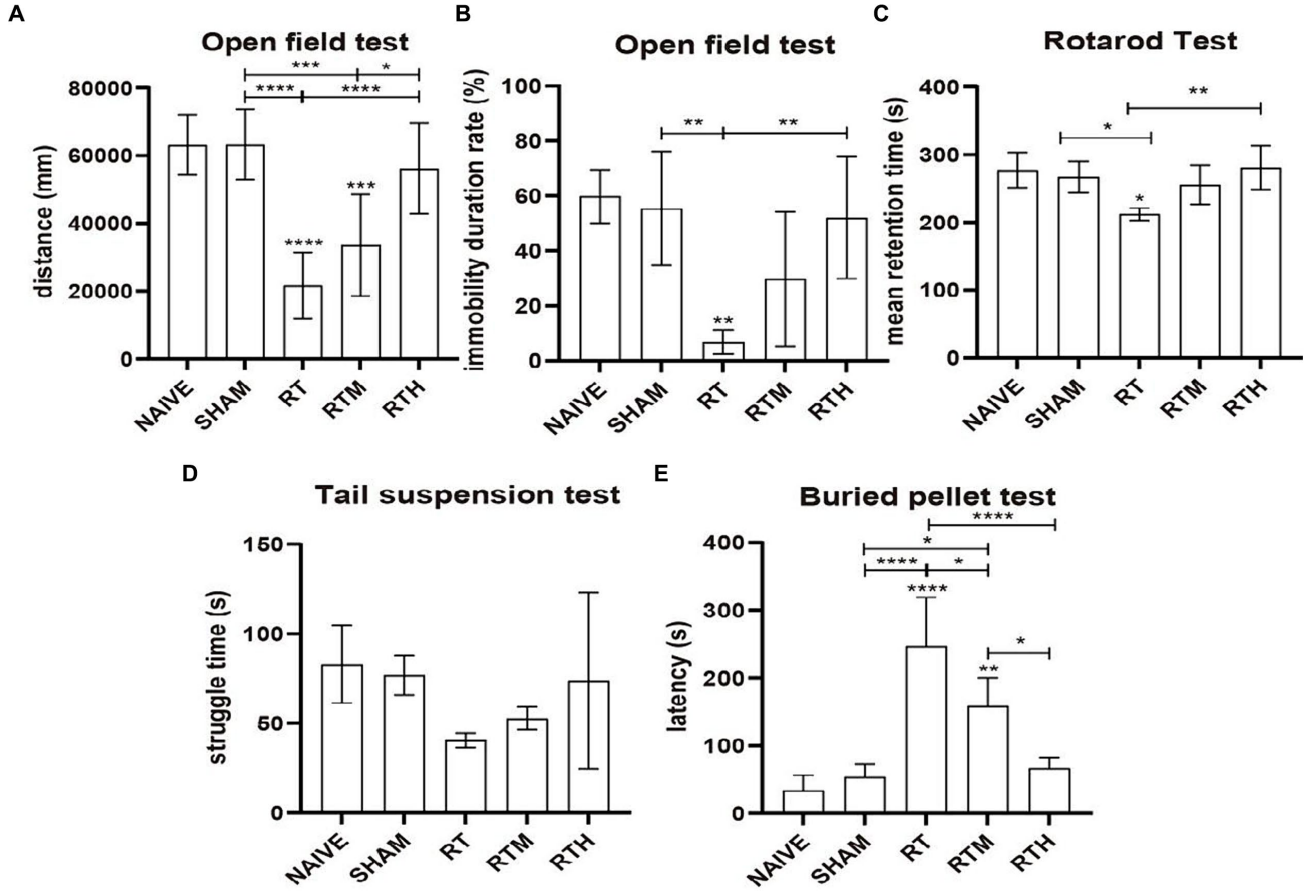
Behavioral tests. Asterisks indicate significant differences after performing all multiple comparison procedures in pairs **(A)**, total movement distance in open field test; **(B)**, immobility duration rate in open field test; **(C)**, the mean residence time in rotard test; **(D)**, struggle time in tail suspension test; **(E)**, the time to discover and eat pellets in buried pellet test. (Holm-Sidak or Dunn’s method; *p < 0.05, **p < 0.01, ***p < 0.001, ****p < 0.0001).

PD rats were subjected to an open field to assess their general motor and anxiety-related behaviors, as shown in Figures 1A,B. Compared with the control group, the central region residence time and total movement distance were significantly reduced in the RT group (*p* < 0.0001). Following NBP treatment, the distance of movement and the length of stay in the central area increased significantly (*p* < 0.0001).

The Rotarod Test was used to assess coordination in rats. As shown in Figure 1C, the mean residence time of the RT group was significantly reduced compared to the control group (*p* < 0.05). Following NBP treatment, the mean residence time was significantly longer (*p* < 0.01).

A tail suspension test was used to assess anxiety-related behavior. Figure 1D shows that the RT group had significantly less struggle time than the control group. Following NBP treatment, the struggle time increased significantly.

As shown in Figure 1E, a buried pellet test was used to assess olfactory behavior. The time to discover and eat pellets was significantly increased in the RT group compared to the control group (*p* < 0.0001). Following treatment with NBP, the time was significantly reduced (*p* < 0.0001).

### 3.2 The result of olfaction improvement

#### 3.2.1 Differentially expressed proteins

**Figure 2:**
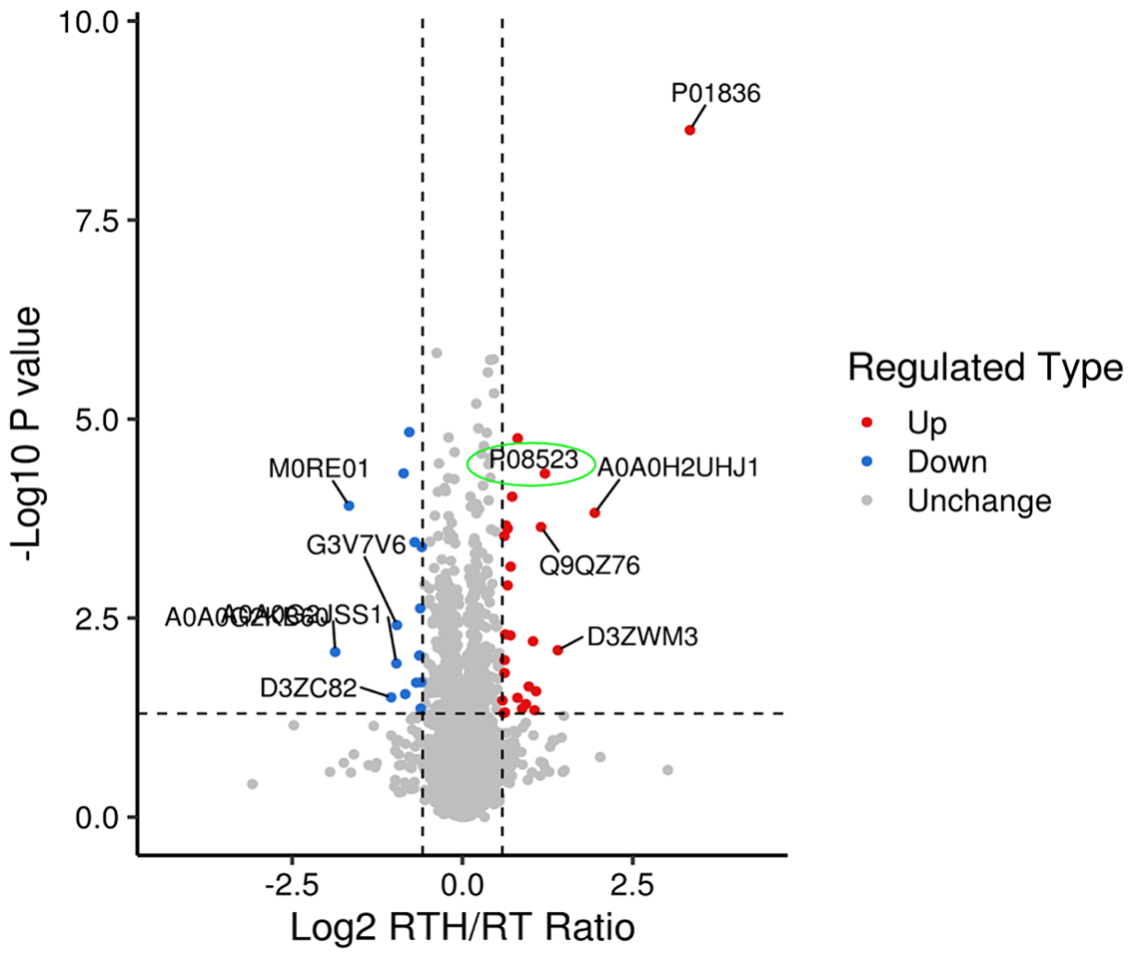
Differential protein volcano map (green color circles the olfactory marker P08523).

Olfactory dysfunction is a crucial non-motor disorder in PD (12). There are few existing studies on the topic (13), and the mechanism of improvement of olfactory dysfunction is unclear. Studying how NBP improves rotenone-induced olfactory sensation in rats with PD may provide us with new ideas for treating PD, for which we did proteomics research. A total of 6,292 proteins and 48,175 unique peptides were identified. The fold change (FC) is used to indicate the difference in the content of all detected proteins, and FC > 1.5 or < 1/1.5, *p* < 0.05. As such, differential protein screening was performed. Compared with the MA group, there were 76 differentially expressed proteins in rat brain tissue after modeling, of which 44 were upregulated and 32 were down-regulated. Following treatment with high-dose NBP, there were 42 differentially expressed proteins compared to the model group, of which 26 were upregulated and 16 were down-regulated. Expressed by a volcano plot (Figure 2), the ordinal number is the significant *p* value of the difference (logarithmic conversion with base 10), the negative number is the fold (logarithmic conversion with base 2), *p* > 0.05 is below the vertical line on the vertical axis, and FC < 1.2 is left of the vertical line on the horizontal axis. Blue dots indicate significantly downregulated proteins, red dots indicate significant up-regulation, and gray dots indicate no difference. Among them, the olfactory marker (P08523) showed a significant upregulation difference in the RTH group compared with the model group.

### 3.3 Verification of top-expressed proteins by RPM

**Figure 3:**
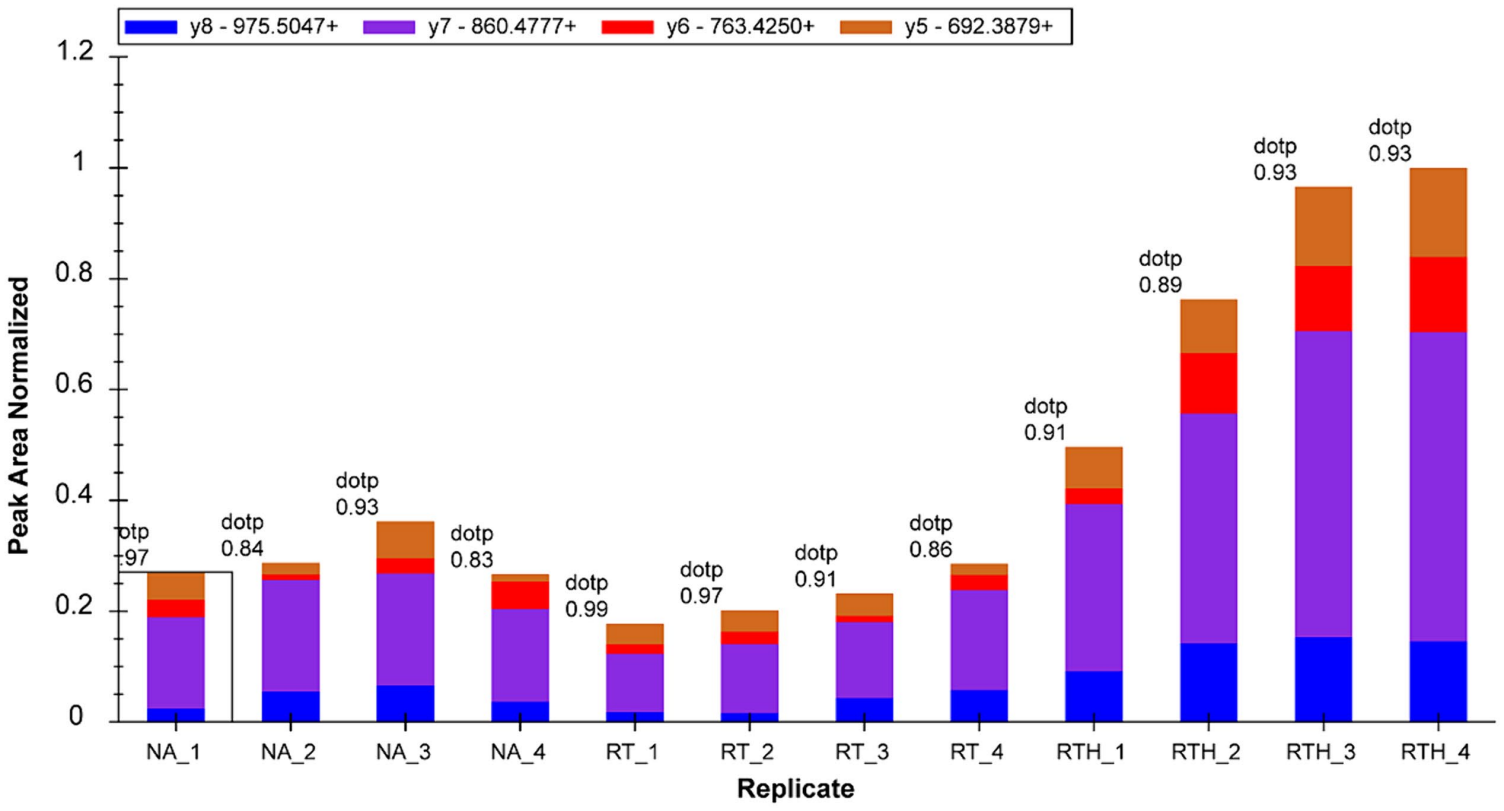
Fragment ion peak area distribution of peptide QLLDPAAIFWR (corresponding to olfactory marker) in 12 samples.

**Figure 4:**
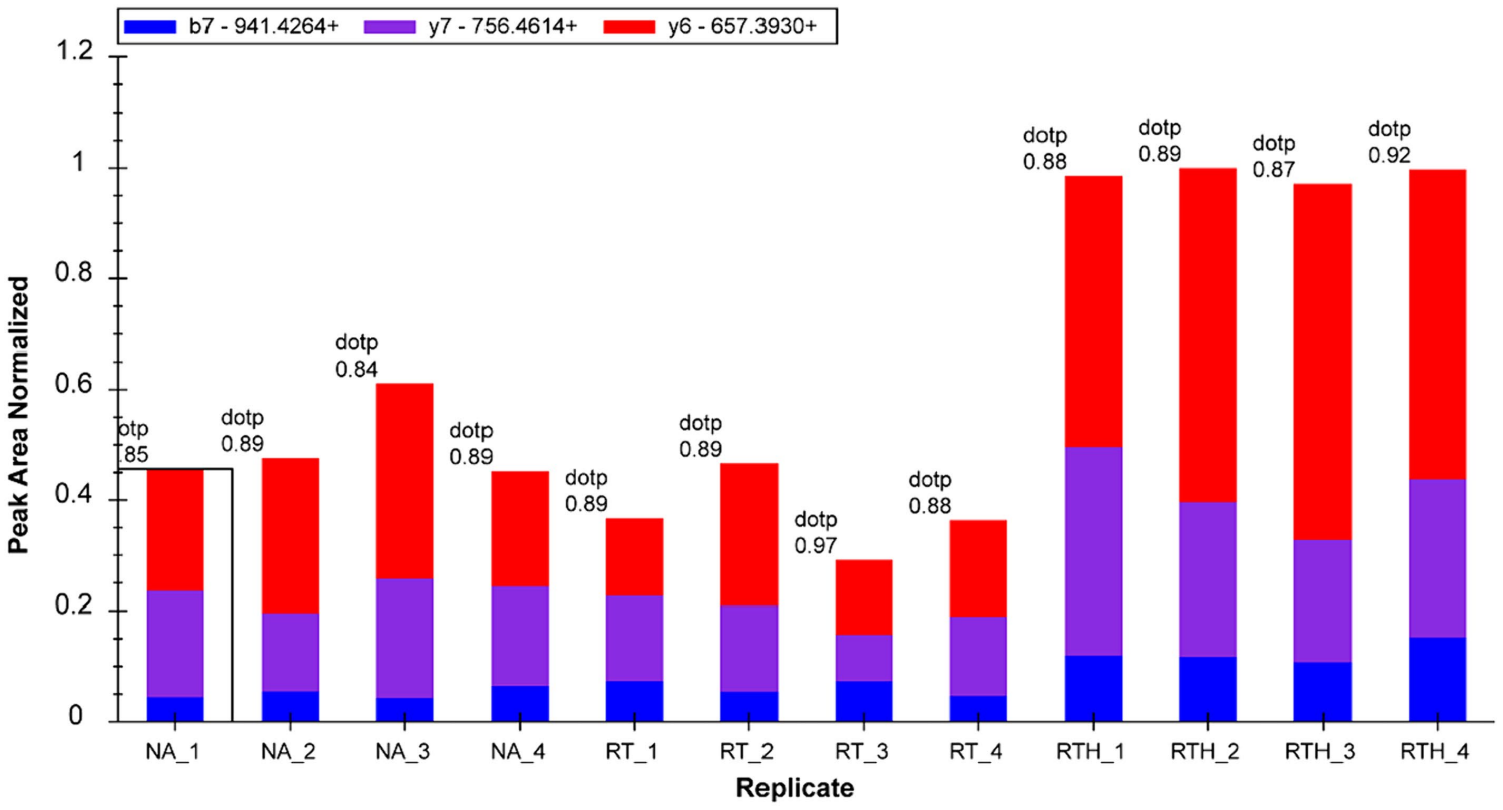
Fragment ion peak area distribution of peptide LQFDHWNVVLDKPGK (corresponding to olfactory marker) in 12 samples.

We selected 22 target proteins for PRM quantification and quantified 16. Among them, olfactory markers were at least 2-fold upregulated in the RTH group compared to the model group, which is manifested in two peptide chains of QLLDPAAIFWR (Figure 3) and LQFDHWNVVLDKPGK (Figure 4).

## 4 Discussion

NBP is a drug extracted from celery seed oil and its chemical formula is C_12_H_14_O_2_ and molar mass is 190.24 g/mol (14) and it benefits anti-inflammatory, anti-oxidative stress, blood–brain barrier protection, cerebral microcirculation improvement, and angiogenesis promotion (10). Since 2002, NBP has been approved in China for the treatment of acute ischemic stroke patients and is now widely used clinically with reliable safety (15). Basic studies have shown that NBP has a therapeutic effect on PD (11). A meta-study of NBP for PD involving 173 patients found that only three treatment-related adverse events occurred in patients treated with NBP and disappeared immediately after discontinuation (14). This meta-analysis demonstrated that NBP is safe and effective in improving motor symptoms and slowing progression in PD patients over the course of 6 months of treatment. Notably, studies have shown that NBP can alleviate motor deficits by inhibiting iron deposition, oxidative stress, and ferroptosis in the substantia nigra (16). Other studies showed that NBP treatment significantly ameliorated motor deficits in the substantia nigra of LPS-induced PD mice, reduced microglial activation, reduced nuclear α-synuclein deposition, and increased the survival rate of TH-positive cells (17). Together, these findings suggest that NBP may exert its therapeutic effects by reducing microglial activation in a mouse model of PD (18). In addition, LPS-induced dopaminergic neurodegeneration was alleviated after NBP treatment, as measured by tyrosine hydroxylase-positive cells (19). In a rodent model, 2 weeks of treatment with NBP improved apomorphine-evoked rotation by 48%, and rescued dopaminergic (DA) neurons by 30% and striatal DA terminals by 49%. Furthermore, NBP upregulated vesicular monoamine transporter 2 gene expression *in vitro* and *in vivo*. In conclusion, NBP may protect DA neurons by reducing oxidative stress (18). Moreover, NBP can exert dopaminergic neuroprotection by inhibiting microglia-mediated neuroinflammation, suggesting that NBP has a good therapeutic effect on PD (20). Thus, NBP may be a new PD treatment drug (21). It is believed that more human clinical trials will be conducted to verify the safety and effectiveness of NBP in the treatment of PD in the near future. Therefore, this article aims to explore the mechanism and pathway of olfactory dysfunction in PD and provide new ideas and methods for the treatment of PD.

Many studies have confirmed the therapeutic effect of NBP on PD. However, there has never been evidence that NBP can directly improve the olfactory perception of PD. Notably, the improvement effect of NBP on olfactory perception has been controversial. The present study found that NBP treatment of rotenone-induced Parkinson’s rats improved the motor symptoms of PD and the expression of olfactory marker proteins. From the buried pellet test, the time for rats to discover and eat pellets was significantly increased in the RT group compared to the control group (*p* < 0.0001). Following treatment with NBP, the time was significantly reduced (*p* < 0.0001). From proteomics research, the olfactory marker (P08523) and the olfactory marker (P08523) showed a significant upregulation difference in the RTH group compared with the model group. PRM quantification results showed that olfactory markers were at least 2-fold upregulated in the RTH group compared to the model group, indicating that NBP may improve the olfactory impairment in PD. Olfactory marker protein (OMP) plays an important role in sensory experience, especially in olfactory experience (22). Additionally, olfactory sensory neurons (OSNs) expressing the same olfactory receptor (OR) are clustered in the same olfactory glomerulus in the main olfactory bulb. Olfactory marker protein (OMP) is a key marker of mature OSN, and its loss is related to OSN signal transduction and odor recognition defects (22). OMP is a protein long known to be expressed in mature olfactory receptor neurons and responsible for controlling proper cAMP homeostasis and dynamics (23). Notably, OMP buffers cAMP and regulates camp-gated channel activity during sensory stimulation, maintaining neuronal firing during odor source search (23). Furthermore, OMP modulates the dynamic range of olfactory receptor neurons in an odor receptor-dependent manner to allow concentration-dependent odor coding (24). Recent studies have also confirmed that in persistent anosmia after +COVID-19 infection, the relative reduction in mature OMP neurons can explain the hyposmia (25).

Olfactory dysfunction is an early marker of PD (26). However the neural substrates of hyposmia are largely unknown. Most attempts to explain olfactory dysfunction in common neurodegenerative diseases have focused on neuropathological markers, such as extracellular amyloid-beta-containing plaques, intracellular neurofibrillary tangles that abnormally phosphorylate tau, or α-synuclein aggregates that makeup Lewy bodies and Louis neuritic processes (27). It has also been proposed that the olfactory epithelium, olfactory bulb and/or olfactory cortex are damaged, and even the centrifugal neuroregulatory system, such as the cholinergic system, is involved (28). The accumulation of misfolded α-synuclein in olfactory-related brain tissues such as the olfactory bulb is still the mainstream view of olfactory dysfunction in PD (29). α-synuclein is the major filamentous component forming Lewy bodies and Lewy neurites (29). The content of dopamine is the highest in the SN-striatum pathway of PD (30). Consequently, a decrease in dopamine function can lead to PD and determine the participation in olfactory processing and olfactory ability (31). Furthermore, some studies have found that in PD patients, serotonin is decreased in the olfactory bulb and olfactory system. Moreover, Lewy pathology is found in the median septal nucleus, indicating that changes in serotonin may be related to OD in PD (32). Therefore, our next research goal will explore whether NBP improves olfactory dysfunction through the above pathways.

## 5 Conclusion

Through proteomics and protein quantification methods, we preliminarily determined that NBP can improve the olfactory impairment of PD, providing new evidence and ideas for treating PD olfactory dysfunction.

## Data availability statement

Anonymized data not published in the article can be made available upon reasonable request from the corresponding author. The raw data sets are available in the publicly accessible repository Dryad at: https://datadryad.org/stash/share/qrjd-vsVzLaWPbWrn0uFaXuQPJcRxluevI9erDDVkeg.

## Ethics statement

The animal experiment was reviewed and approved by the Animal Ethics Committee of the Affiliated Hospital of Shandong University of Traditional Chinese Medicine, approval number: AWE-2022-014. The experiment was conducted in accordance with the local legislation and institutional requirements and all aspects of the experiment followed the 3R principle. Anesthesia and analgesia were performed by intraperitoneal injection of pentobarbital sodium 320 mg/kg.

## Author contributions

JW: Writing – original draft, Methodology, Conceptualization. TW: Writing – review & editing, Funding acquisition. HL: Writing – original draft, Methodology, Conceptualization. XX: Writing – original draft, Methodology, Conceptualization. CW: Writing – original draft, Software, Data curation. DL: Writing – original draft, Visualization, Investigation. YZ: Writing – original draft, Validation, Software. MS: Writing – original draft, Supervision.
